# The “gray zone” in pediatric end-of-life care: bioethical and medico-legal reflections

**DOI:** 10.3389/fped.2025.1599837

**Published:** 2025-06-25

**Authors:** Roberto Scendoni, Francesco De Micco

**Affiliations:** ^1^ Department of Law, Institute of Legal Medicine, University of Macerata, Macerata, Italy; ^2^ Research Unit of Bioethics and Humanities, Department of Medicine and Surgery, Università Campus Bio-Medico di Roma, Roma, Italy; ^3^ Department of Clinical Affair, Fondazione Policlinico Universitario Campus Bio-Medico, Roma, Italy

**Keywords:** palliative care, end-of-life decision, bioethics, pediatrics, death

## Abstract

Palliative care for children with incurable diseases represents one of the most complex challenges in pediatric medicine. It requires a delicate balance between continuing potentially ineffective therapies and ensuring comfort and dignity during the terminal phase. Decisions involve both the family and the medical team, with a particular focus on the “gray zone,” where prognostic uncertainty makes it difficult to determine the most ethical course of action. According to the WHO, palliative care aims to improve the quality of life for both the child and their family, addressing not only physical pain but also psychological and social issues. This article explores the bioethical and medico-legal implications surrounding end-of-life care, parental decision-making autonomy, and the need to always prioritize the child's best interests, while respecting their dignity and values.

## 1 Introduction

Palliative care for children with an incurable disease is one of the most delicate areas of pediatric practice. The peculiarities surrounding this end-of-life phase are mainly linked to the tragic nature of a child's death, despite awareness of the terminal diagnosis; for parents it is unnatural to outlive a child, and this type of loss is a devastating experience. It can also be emotionally challenging for caregivers. In neonatal death, the clinical complexity of this event inevitably involves the entire network of care for the newborn. Often the parents or caregivers themselves are not united in establishing when it is time to stop making desperate attempts to find a cure and when instead it is time to prioritize comfort, dignity, and peace for the child and their family. When faced with complex situations where decisions must be made about the care and resuscitation to be provided, disputes may arise among the healthcare team; and it may not be clear which is the right path to follow (1). It is important to emphasize that pediatric palliative care is not limited to end-of-life management, but rather represents a comprehensive approach to caring for children with chronic, complex, or incurable conditions, starting from the moment of diagnosis. PPC aims to improve the quality of life of both the patient and their family by preventing and relieving suffering through addressing physical, psychological, social, and spiritual needs. This type of care can be integrated early alongside curative treatments and continues throughout the course of the illness, supporting better symptom management, providing consistent support to the family, and facilitating complex decision-making processes. Reducing PPC to end-of-life care alone risks limiting its scope and effectiveness, particularly within a patient- and family-centered care perspective. To aid the decision-making process in such contexts, it is helpful to consider three fundamental questions: (a) Is the treatment mandatory? (b) Would the treatment be unreasonable and should therefore be with-held? and (c) Should treatment be offered as an option, in view of high prognostic un-certainty? In this last situation, where a patient's outlook is unclear, doubts arise as to what course of action will provide the most ethical outcome. This has been called the “gray zone” (2).

## 2 Access to pediatric palliative care

According to the World Health Organization (WHO), palliative care is concerned with preventing and alleviating the suffering of an adult or child patient with a life-threatening illness (3). Addressing suffering includes taking care of psychosocial and spiritual problems associated with the illness as well as the physical symptoms. Palliative care also extends to the patient's family members in terms of providing them with support to cope with such a difficult time. Palliative care is characterized by a complex and articulated structure, based on three fundamental dimensions: clinical objectives, an integrated and continuous approach, and a multidisciplinary organizational model (4) (Table 1).

**Table 1:** Main thematic domains and corresponding core principles in pediatric palliative care.

Thematic area	Key principles of pediatric palliative care
Clinical Goals	– Identify, assess, and treat pain and other symptoms as early as possible – Improve quality of life for patients and families, maintain dignity and comfort – May positively influence the course of the illness – Offer alternatives to disease-modifying and life-sustaining treatments of questionable benefit near the end of life – Never intentionally hasten death, but ensure adequate comfort in accordance with the patient's values – Support those living with long-term physical, psychological, social, or spiritual sequelae of serious illness or its treatment
Integrated and Continuous Approach	– Support the patient and family throughout the course of illness – Begin early in the course of illness, alongside life-prolonging treatments – Be incorporated into national health strategies to complement prevention, early diagnosis, and treatment of serious illness
Organizational and Multidisciplinary Model	– Involve a multidisciplinary network of healthcare workers (primary care providers, generalists, and specialists with varying levels of palliative care training) – Be available both at home and across all levels of the healthcare system – Promote the active involvement of community members

Geopolitical situations, socioeconomic conditions, and culture are all factors that can influence the type and severity of suffering. Children and their families in low- and middle-income countries (LMICs) often endure unhealthy social conditions. They also typically have less access to disease prevention, diagnosis, treatment, social support, and specialized services of many kinds than children in high-income countries (HICs). For example, many children have limited or no access to cancer chemotherapy, radiation therapy, oncologic surgery, and pediatric intensive care. Palliative care must never be seen as a replacement for disease prevention, treatment, or critical care, and palliative care workers have a duty to advocate for them wherever they are unavailable (5, 6). Furthermore, the same standards of palliative care should be universally accessible. In LMICs pediatric end-of-life care is inadequate due to the lack of economic resources. The reduced availability of drugs to alleviate pain and suffering makes it woefully difficult to implement a personalized palliative care plan (7). Scientific evidences confirm that access to pediatric palliative care (PPC) in low- and middle-income countries (LMICs) is extremely limited, despite the fact that the need is greater than in high-income countries. It is estimated that over 98% of children in need of palliative care live in LMICs, but in many of these settings less than 5%–10% actually receive adequate care. Major barriers include a shortage of trained health workers, limited access to essential medications-particularly opioids for pain control-and the absence of dedicated and integrated facilities in national health systems. However, some good examples, such as the implementation of PPC programs in Colombia, show that with proper institutional and organizational support, access to these services can be significantly improved. Inequality in pediatric palliative care ultimately reflects a profound global inequality in health equity, which is strongly correlated with a country's income level (8–10). The scope of palliative care is not limited to problems associated with incurable or life-threatening diseases. Globally, in both HICs and LMICs, children with severe physical disabilities carry a heavy burden of suffering. There is a diverse range of disabilities, including those due to traumatic injury, congenital anomalies, and genetic disorders. However, palliative care approaches can help to alleviate common types of suffering experienced by children with disabilities, for example pain and social isolation or stigmatization (11). Depending on the particular condition, other chronic physical or psychological symptoms may be reported. Moreover, an individual's inability to feed or wash themselves, walk or use the toilet without assistance imposes physical, financial, and emotional burdens on the family, especially among the rural poor (for whom palliative care providers may be the only source of support) (12). Thus, based on WHO's recent recommendations (13):

▪ Palliative care for children is the active total care of the child's body, mind, and spirit, and involves giving support to the family.
▪ It begins when illness is diagnosed and continues regardless of whether a child receives treatment targeted at the disease.
▪ Health providers must evaluate and alleviate a child's physical, psychological, and social distress.
▪ Effective palliative care requires a broad multidisciplinary approach that includes the family and makes use of available community resources; it can be successfully implemented even if resources are limited.
▪ It can be provided in tertiary care facilities, in community health facilities, and even in children's homes.

## 3 Liability for the decision-making process in the gray zone

In the context of pediatric palliative care, the decision-making process cannot be reduced to a simple attribution of responsibility on “who has the last word”, but must be understood as a shared path, which clinicians and families undertake together. It is a relational and communicative path that is built over time, based on mutual trust, active listening and recognition of the emotions, values and expectations of all the subjects involved. The complexity of clinical situations and the delicacy of choices—often linked to the prognosis, the quality of life of the child, and the proportionality of care—require a co-construction of decisions, in which healthcare professionals offer technical skills and ethical perspectives, while families bring their deep knowledge of their child, of the emotional and cultural context in which they live. Pediatric palliative care, precisely because of its holistic and personalized nature, promotes this model of therapeutic alliance, in which choices mature gradually, respecting the times, uncertainties and emotional experience of each person. In this sense, it is not just about “deciding”, but about accompanying, creating a space in which care itself is a shared act of humanity and responsibility.

In conscious adults, there is currently a consensus that patients themselves, exercising their autonomy, have the right to make the decision of whether to accept or refuse treatment after having been informed of the options. In the case of newborn babies or infants who lack autonomy, the decision lies with their immediate family, i.e., the parents, and the health care team. This is a complex issue. Nowadays, parents are typically granted the right to decide for their children, since it is widely accepted that they are in the best position to define what is in their child's best interest, except of course in cases of abandonment, neglect, or abuse. It is important to recognize, at the same time, that this right does not imply parental obligation, given that assuming responsibility in this way causes anguish and, eventually, guilt. In these situations, health care providers should empathize, make informed recommendations, seek agreement, and avoid placing the burden of the decision on the parents. However, in this gray zone, sometimes none of the available options seem right. In the case of patients with severe conditions that affect their quality of life or are life-threatening, the fundamental options are to continue life-sustaining therapies or to allow death. A decision needs to be made after reflection through informed discussion among the parties involved. It is rightly said that the worst decision is the one not taken. It would be a potentially devastating non-decision to let things run their course without facing the problem, without considering how to improve the patient's care and living (or dying) conditions (14). It is interesting that both options (continuing therapy or withdrawing life-sustaining measures) are ethically defensible, even if some may disagree as to which is the “right” path (15).

Making decisions about whether to use, withhold, or withdraw disease-modifying or life-sustaining treatments of questionable value for a child can be especially complex. Parents often find it more difficult to understand or accept the poor prognosis of a child than of an aged family member. Clinicians, too, may find it challenging to weigh up the relative benefits and burdens of interventions for pediatric patients who are unable to speak for themselves. Furthermore, approaches to decision-making in this context vary between cultures, and sometimes there can be disagreement even within the same family. Gentle but determined efforts should be made to try to understand the child's perspective, wherever possible.

## 4 Bioethics and medico-legal insights into the end-of-life phase in children

Based on recent studies conducted on the topic, children in the following groups are more likely to receive palliative care (16, 17):

(1) “Life threatening” conditions for which therapeutic treatment may be feasible but may fail. Where access to pediatric palliative care services may be necessary when treatment fails, children in long-term remission or those who have received successful therapeutic treatment are not included (e.g., irreversible organ failure, cancer)
(2) Cases in which premature death is inevitable, and where long periods of intensive treatment are needed to prolong life and enable child patients to participate in normal everyday activities (e.g., cystic fibrosis)
(3) Progressive conditions with no curative treatment options, where treatment is exclusively palliative and may commonly be extended for many years (e.g., mucopolysaccharidosis, muscular dystrophy)
(4) Irreversible conditions, causing severe disability leading to susceptibility to health complications and the likelihood of premature death (e.g., severe cerebral palsy, severe asphyxial sequelae).

Every child and family should be helped to make decisions about end-of-life planning and should be continually supported in all aspects of this planning. All this in accordance with the fundamental principles of the Convention on the Rights of the Child (CRC) (18):

(a) Non-discrimination (art. 2): the rights established by the Convention must be guaranteed to all minors, without distinction of race, sex, language, religion, opinion of the child/adolescent or parents.
(b) Best interests (art. 3): in every law, provision, public or private initiative and in every problematic situation, the interest of the child/adolescent must have priority.
(c) Right to life, survival and development of the child and the adolescent (art. 6): States must commit the maximum available resources to protect the life and healthy development of children, also through international cooperation.
(d) Listening to the opinions of the minor (art. 12) provides for the right of children to be heard in all decision-making processes that concern them, and the corresponding duty, for adults, to take their opinions into adequate consideration.

The gray zone in end-of-life decision-making for children is delimited by two distinct phases, each of which raises bioethical and medico-legal issues related to care: before death and at the time of death.

### 4.1 Before death

The critical issues of this phase are essentially linked to the complexity in making decisions and managing symptoms. The first objective is to ensure adequate communication as this is central to the relationships between the network of health and social care professionals, the family, and the child. Particular attention should be given to: body language and non-verbal communication skills; the ability to listen and remain silent; asking open-ended, targeted questions; and building a relationship of trust that is always respectful of the other. The child's family members and, whenever possible, the dying child should be involved and supported in making decisions, while keeping in mind that they can often change their point of view. In the context of shared care planning, the ability to fully commit is essential to ensure that trust is established between the team, the child and their family (19).

The main difficulties include insufficient time to reflect, clinical uncertainty, changes in personnel, and disagreements between teams and families. On the contrary, clear, empathetic, and structured communication, along with a strong relationship of trust, is crucial to foster shared decisions. Parents want to be actively involved, but excessively technical or ambiguous medical language can limit their participation. In intensive care, the decision-making role is often centralized in doctors, while the contribution of nurses remains marginal. The most complex conversations occur in the presence of prognostic uncertainty, and require refined communication strategies that balance information, empathy, and support. Some studies propose structured tools to analyze and improve the dialogue between caregivers and families, and emphasize the importance of clinical and ethical training of operators. Finally, there is a clear need for stable teams, shared protocols, and a greater understanding of the dying process to ensure coherent, respectful, and humanely sustainable choices (20–25).

At this stage, parents of a newborn may ask what their child's fate will be or how long they will survive. The role of the health care team must be to answer honestly, based on evidence from literature and avoiding hypotheses that are not in line with reality. Furthermore, the place where the child and their family prefer to be assisted at the end of life should be agreed upon at this stage, although this can change based on individual needs.

For children who require end-of-life care at home, all of the following should be guaranteed: access to 24-hour counselling from a pediatric palliative care specialist; 24-hour pediatric nursing care; home visits by a palliative care specialist for symptom management; practical support with necessary medical devices (e.g., oxygen, aspirator, enteral nutrition, intravenous therapies, etc.); and advance prescriptions for children who are more likely to develop certain symptoms (e.g., constipation in the case of regular administration of opioids, etc.) (26). Inadequate management of end-of-life symptoms can also cause difficulties in the parents' grieving process. Hence the need to provide an adequate response to these symptoms as they evolve. Sometimes parents fear that the use of analgesic drugs can somehow hasten death; the caregiving team must on the one hand find the right balance in medication titration to obtain the desired effect in proportion to the severity of symptoms, and on the other hand communicate clearly with parents who can also be involved in assessing the evolution of symptoms (27).

### 4.2 At the time of death

In children with life-limiting or life-threatening conditions, death may occur suddenly or not; the child may be connected to machinery or receive an infusion of vasoactive drugs and/or hydration. The termination of life-sustaining treatment (which includes forgoing artificial nutrition and hydration) may be ethically justifiable when the burden of treatment exceeds the benefits for the child (28). Some research has demonstrated that parents are more likely to accept the situation when they are reassured that their child is dying because of their illness and not because of the withdrawal of treatment (29).

Particular ethical questions arise in the issue of gaining consent for organ donation from children. When caring for the family and the child who will be an organ donor, health care providers and support services can influence parents' decisions about organ donations (30, 31). By recognizing that families see grief not as a problem to be solved, but as a process to be experienced, health care providers can leverage both internal hospital resources and community networks to support and surround families as they navigate this difficult time. Obviously, the question arises as to whether the principle of self-determination and choice must always be preserved in parents who consent to donation. Is it ethically justifiable for social and care networks to attempt to impact their decision (32)?

Bioethicists and medico-legal experts are currently debating this issue.

Paediatric palliative care, according to internationally recognised best clinical practices, requires clear, empathetic and family-centred communication, supported by an adequately trained multidisciplinary team. It is essential to guarantee 24-hour home access with dedicated operators, in a context of shared and flexible planning with respect to care preferences. Palliative care does not replace curative treatments, but integrates them from the beginning, ensuring global and continuous care of the child and his family (33, 34).

## 5 Medico-legal issues

According to the Italian National Bioethics Committee, the child's best interest is the main criterion for reaching clinical decisions, taking into consideration their pain, suffering, and dignity. It is essential to avoid clinical overkill by relying on objective data and ensuring the best quality of treatment available (35).

Unfortunately, there is no agreement in the international literature on the meaning of “futile treatment”, so much so that in the English-speaking context, this term is little used. Emphasis has been placed on the concept of futility in relation to interventions or treatment, considered ineffective. However, futile treatment is not necessarily ineffective, and the American Medical Association's Code of Ethics (36) states that there is no universal definition of futility, as it depends on the specific values and goals of each patient and on their individual circumstances (36).

This makes decision-making in this “gray zone” even more sensitive. Therefore, a shared definition of therapeutic futility is indispensable, as a prerequisite for assessments which may be clouded by the contrast between an objective medical and scientific judgment, albeit partly probabilistic, and a patient's subjective perception.

First, it is necessary to go beyond the term of therapeutic overkill to more correctly speak of therapeutic obstinacy. In the medical field, the intent of health care providers is always aimed at patient care. Even when a physician persists in interventions or treatments that may seem unwarranted or ineffective, it is not out of a desire to cause harm, but rather out of a sense of responsibility, hope or professional obligation to do everything possible for the patient.

Indeed, a therapy can be said to be obstinate when it is documented to be ineffective in relation to the goal, when it may involve high risk and/or special burdens for the patient with additional suffering, and when the exceptional nature of the means employed is clearly disproportionate to the goals of the specific condition (37).

The inadequacy of technical and medical care must be assessed in relation to the fundamental objectives of healthcare, namely to preserve the patient's life and health, as well as to improve their quality of life. To achieve these objectives, it is essential to rely on objective scientific and clinical data, which take into account various aspects: the severity of the disease, its progression over time, the therapeutic alternatives available, and the realistic prospects of survival, recovery, or risk of death. The extent of the risks associated with a medical intervention must be carefully assessed in relation to the severity of the pathology and the clinical urgency of the situation. In particularly serious and urgent clinical conditions, the acceptable risk threshold tends to be higher, since the need to intervene outweighs the potential dangers associated with the treatment. However, this risk threshold should not be determined arbitrarily but based on solid criteria shared by the scientific community. It is essential that these decisions are informed by up-to-date guidelines, validated clinical data, and expert consensus, to ensure that the chosen approach is as appropriate as possible to the specific conditions of the patient, minimising risks without compromising the chances of a favorable outcome. To assess the severity of a medical treatment, the individual patient's perception of the extraordinary nature of the intervention must be considered, in the context of their subjective overall experience of therapy. Finally, assessment of the disproportionate use of diagnostic, therapeutic, and life-sustaining means also depends on the poor availability, difficult findability, and technical use of the means. Such assessments are particularly complex in the case of rare diseases, where the diagnosis and prognosis are highly unpredictable, mostly probabilistic, and never certain (38–41).

In any case, the moral prohibition against futile treatment must never result in abandonment of the child. Physicians have an absolute duty to provide appropriate treatments and support, which include both technological and pharmacological principles and palliative care, accompanying the child through the dying process. This may include, when necessary, the use of continuous deep sedation, combined with pain therapy, to ensure a dignified end without suffering (42).

Therapeutic obstinacy is distinguished from therapeutic insistence, whereby the physician is committed to prolonging life-sustaining therapies even for a long time, in the face of prognostically unpredictable situations. In contrast to therapeutic obstinacy, therapeutic insistence is the result of prudential medical conduct and, therefore, at least in general, ethically positive (43).

Having circumscribed the scope of action and defined and differentiated the concepts of therapeutic obstinacy and therapeutic insistence, it is necessary to define who makes the decisions.

It seems fair that the process of shared care planning should involve the medical team and parents, ensuring space and time for communication. But there is more to it than this. Depending on age, the young patient should also be involved in the care and treatment plan. Although consent to the medical act should be sought from those exercising parental responsibility or guardianship, it is necessary to provide the child patient with the appropriate information and rationale to help prepare them for the proposed treatment. Moreover, minors should be reassured that the health care team is committed to listening to their requests and preferences and will provide explanations and help, whenever necessary.

Bioethical evaluation and medico-legal judgment based on objective medical assessment and considerations of the patient's subjective perceptions represent a scientifically sound approach to the legalization of termination of life (44).

However, there is risk of abuse in the application of the Groningen Protocol, under which lethal injection can be given to infants with spina bifida following an assessment of incurable and intolerable suffering. Decisions about which infants are candidates for euthanasia under the protocol may be highly subjective, based on perceptions of quality of life and predictions that may not be accurate. In addition, families may be influenced to choose euthanasia for their newborn due to social, economic, or cultural pressures, rather than an objective assessment of the child's best interest (45). In sum, these issues raise serious ethical and legal questions about the future of such practices.

## 6 Conclusions

The last hours, days, and sometimes weeks in the life of a child affected by an incurable disease represent a profound challenge for the family and for the professionals who are part of the child's care network. In order to adopt the best care strategies in the end-of-life phase in children, every individual case needs to be analysed according to its specificities. In the gray zone, some choices must be made jointly by health professionals and by the relatives of the child. Such decisions are neither easy nor univocal (46). The fact remains that in deciding which interventions to implement, those who deal with pediatric palliative care daily reiterate the importance of starting from the needs of the child and their family (47).

## Data Availability

The original contributions presented in the study are included in the article/Supplementary Material, further inquiries can be directed to the corresponding author.
